# Learning from Nature: Pregnancy Changes the Expression of Inflammation-Related Genes in Patients with Multiple Sclerosis

**DOI:** 10.1371/journal.pone.0008962

**Published:** 2010-01-29

**Authors:** Francesca Gilli, Raija L. P. Lindberg, Paola Valentino, Fabiana Marnetto, Simona Malucchi, Arianna Sala, Marco Capobianco, Alessia di Sapio, Francesca Sperli, Ludwig Kappos, Raffaele A. Calogero, Antonio Bertolotto

**Affiliations:** 1 Regional Centre for Multiple Sclerosis (CReSM) and Clinical Neurobiology, Azienda Ospedaliera Universitaria San Luigi Gonzaga, Orbassano, Italy; 2 Departments of Biomedicine and Neurology, University Hospital Basel, Basel, Switzerland; 3 Genomics and Bioinformatics Unit, Department of Clinical and Biological Sciences, Azienda Ospedaliera Universitaria San Luigi Gonzaga, Orbassano, Italy; Julius-Maximilians-Universität Würzburg, Germany

## Abstract

**Background:**

Pregnancy is associated with reduced activity of multiple sclerosis (MS). However, the biological mechanisms underlying this pregnancy-related decrease in disease activity are poorly understood.

**Methodology:**

We conducted a genome-wide transcription analysis in peripheral blood mononuclear cells (PBMCs) from 12 women (7 MS patients and 5 healthy controls) followed during their pregnancy. Samples were obtained before, during (i.e. at the third, sixth, and ninth month of gestation) and after pregnancy. A validation of the expression profiles has been conducted by using the same samples and an independent group of 25 MS patients and 11 healthy controls. Finally, considering the total group of 32 MS patients, we compared expression profiles of patients relapsing during pregnancy (n = 6) with those of relapse-free patients (n = 26).

**Principal Findings:**

Results showed an altered expression of 347 transcripts in non-pregnant MS patients with respect to non-pregnant healthy controls. Complementary changes in expression, occurring during pregnancy, reverted the previous imbalance particularly for seven inflammation-related transcripts, i.e. SOCS2, TNFAIP3, NR4A2, CXCR4, POLR2J, FAM49B, and STAG3L1. Longitudinal analysis showed that the overall deregulation of gene expression reverted to “normal” already within the third month of gestation, while in the post-partum gene expressions rebounded to pre-pregnancy levels. Six (18.7%) of the 32 MS patients had a relapse during pregnancy, mostly in the first trimester. The latter showed delayed expression profiles when compared to relapse-free patients: in these patients expression imbalance was reverted later in the pregnancy, i.e. at sixth month.

**Conclusions:**

Specific changes in expression during pregnancy were associated with a decrease in disease activity assessed by occurrence of relapses during pregnancy. Findings might help in understanding the **pathogenesis of MS** and **may** provide basis for the development of novel therapeutic strategies.

## Introduction

Pregnancy represents a physiological transitory state of immune tolerance to avoid the rejection of the fetus, and is frequently associated with reduced activity of autoimmune diseases, including multiple sclerosis (MS). Natural course studies in MS have shown that the relapse rate during pregnancy, especially in the third trimester, decreases more than under treatment with currently approved first line treatments interferon-beta or glatiramer acetate. During the first three months post-partum an increase of the relapse rate follows before returning to the pre-pregnancy rate [1]–[3]. In general, the lower relapse rate during pregnancy might be due to a pregnancy-associated down-regulation of cell-mediated immunity.

While the etiology of MS is unknown, autoreactive Th1/Th17 cells are thought to play an essential role in the pathogenesis of the disease [4]–[5]. On the contrary, pregnancy results in a shift towards Th2 cytokine profile [6]. Thus, the protective effect of pregnancy in MS might be explained by the antagonistic effects of Th2 response, which inhibit the development of both pro-inflammatory Th1 and Th17 cells [7].

The whole balance among Th1, Th2, and Th17 is regulated by a specific subpopulation of T-lymphocytes, e.g. CD4^+^CD25^+^ regulatory T-cells (T_reg_), which are activated to modulate immune responses to avoid over-reactive immunity [7]. Recently, an increase in CD4^+^CD25^+^T_reg_ has been described in humans during pregnancy [8], [9]. Hence, systemic expansion of CD4^+^CD25^+^ T-lymphocytes may add on the concept of immune system pregnant-related suppression and might support the better clinical MS pregnancy course.

A variety of pregnancy-factors can be involved in this immune shift, including sex hormones [10]–[12], which appear to be involved in regulating the immune response [13]. Estrogens, indeed, are known to exert opposing, bimodal, dose-specific effect to immune response: low levels facilitate a cell-mediated pro-inflammatory immune response, whereas their relatively high levels, such as those achieved during pregnancy, promote anti-inflammatory Th2 responses.

Collectively, the protective effect of pregnancy in MS and the anti-inflammatory effects of estrogens have suggested that hormones associated with pregnancy may exert the beneficial influence on MS. Accordingly, a pilot clinical trial using oral estriol to treat MS patients showed significantly decreased gadolinium enhancing lesions on monthly cerebral MRI [14].

As a whole, however, no one has until now used a molecular large-scale approach to study the pregnancy phenomenon in MS and thus still little is known about the molecular mechanisms behind the adaptive molecular processes of pregnancy. In the present study we aim to use modern high-throughput microarray technology to analyze gene expression profiles during pregnancy in MS patients and compare them to those in healthy pregnant volunteers. We seek to determine the MS-associated genes, which modify their status during pregnancy as a consequence of either changes in expression in the peripheral blood mononuclear cells (PBMCs) or changes in cell composition in the PBMC populations during pregnancy. Such elements will improve the understanding of protective mechanisms in the maternal immunology of pregnancy, as related to the pathogenesis of MS, potentially leading to the development of novel therapeutic strategies for MS.

## Methods

### Ethic Statements

This study was approved by the Ethical Committee of the San Luigi University Hospital (March 2006, approval n°87), and the research was conducted in accordance with the Declaration of Helsinki. All subjects with a desire to become pregnant were potentially included in the study and informed, written consent was obtained from each of them.

### Patients and Controls

In patients with a desire to become pregnant, all disease modifying treatments (DMTs) were withdrawn for at least three months before actively trying to become pregnant. Three women who had an abortion in the first trimester were excluded, while two patients were excluded because they were not clinical homogeneous with the remaining patients: one had a clinical isolated syndrome (CIS) and one had a severe iron deficiency anemia concurrent to MS. Finally, a consecutive sample of 32 patients with clinically definite relapsing-remitting MS [15] was enrolled.

Twenty-one age-matched healthy women were included as controls: 16 of them became pregnant. Pre-pregnancy samples from all of these healthy women were analyzed for gene expression.

Demographic and clinical characteristics of healthy controls and MS patients at baseline are shown in table 1 and table 2.

**Table 1:** Demographic characteristics of the 48 pregnant women.

Category	Subcategory	MS patients	Healthy Controls
***No. of women***		32	16
***Age at the beginning of pregnancy (yrs.)***		33.19±4.15	31.95±3.88
***Months at delivery***	<9 months	2	1
	9 months	30	15

**Table 2:** Clinical characteristics of the 32 pregnant women with multiple sclerosis.

Category	Subcategory	Months	No. of patients	No. of Relapses (No. of patients)	EDSS (median)
Duration of MS before pregnancy		79.54±49.61			
***Before pregnancy (12 mo.)***				16 (15)	1.0
Treatment	IFNβ-1a 30 µg im		11		
	IFNβ-1a 22 µg sc		7		
	IFNβ-1a 44 µg sc		5		
	IFNβ-1b 250 µg sc		3		
	Glatiramer Acetate		3		
	No treatment		3		
***During pregnancy (9 mo.)***	1^st^ trimester			5 (5)	1.0
	2^nd^ trimester			1 (1)	1.0
	3^rd^ trimester			1 (1)	1.0
***After Pregnancy (12 mo.)***				14 (11)	1.5

### Study Design

The subjects were followed in the outpatient's clinic and blood was collected before pregnancy and at the following time points during pregnancy: first trimester (gestational age at sampling 12±1 weeks), second trimester (gestational age at sampling 24±1 weeks), and third trimester (gestational age at sampling 36±1 weeks). Post-partum blood samples were collected in 28 of the 32 MS patients, mostly (25/28) between 3 weeks and 4 months after delivery. In two patients blood was collected 8 months after delivery, and in one patient the post-partum blood sample was obtained 10 months after delivery. Likewise, post-partum blood samples were collected in 14 of the 16 healthy controls, mostly (13/14) between 3 weeks and 4 months after delivery. In one healthy control blood was collected 6 months after delivery. Before-pregnancy samples were obtained from both groups after contraceptive drug withdrawal; similarly, both before- and after-pregnancy samples were obtained from MS patients in a DMTs-free period.

PBMCs obtained from 12 women (7 MS patients +5 healthy controls) were analyzed by oligonucleotide microarray technology. Microarray findings were then corroborated and extended by real time RT-PCR in the same cohorts of subjects, as well as in an independent group of 25 MS patients and 11 healthy controls.

### Total RNA and Protein Extraction

PBMCs were isolated from whole blood by centrifugation over Lymphoprep™ (Axis-Shield, Oslo, Norway); total RNA and protein were extracted with TriReagent® following the manufacturer's instruction (Ambion, Austin, TX). RNA and proteins were stored at −80°C until used.

### Microarray Analysis

Double strand cDNA was synthesized from one µg of total RNA using a cDNA Synthesis System (InvitroGen, Basel, Switzerland) with the T7-(T)_24_ primer. The *in vitro*-labeling kit (Enzo; Farmingdale, NY) was used to transcribe the cDNA into cRNA in the presence of Biotin-11-CTP and Biotin-16-UTP, according to the kit instructions. After purification with the MinElute kit (Qiagen; Hilden, Germany) integrity of the cRNA was assessed by both gel electrophoresis and BioAnalyzer (Agilent Technologies, Palo Alto, CA). Twelve µg fragmented cRNA was then used for hybridization to the Human Genome U133A 2.0 array (Affymetrix; Santa Clara, CA). The array contains 21722 probe sets corresponding to approximately 14564 transcripts. Hybridization and staining were performed as described previously [16], [17].

### Real Time RT-PCR

Total RNA (10 ng/µL) was reverse transcribed using random hexamer primers with the High Capacity cDNA Kit (Applied Biosystems, Foster City, CA). cDNA was used as a template for the real time RT-PCR analysis based on the 5′-nuclease assay, with the ABI PRISM® 7000 Sequence Detection System. For primers and probes, Applied Biosystems' TaqMan® gene expression assays were used. Transcriptional expression was normalized using the housekeeping gene glyceraldehyde-phosphate-dehydrogenase (GAPDH) as reference, in order to avoid differences due to possible RNA degradation/contamination or different reverse transcription efficiencies. Relative expression levels of targets were calculated by the comparative cycle threshold (C_t_) method outlined in user bulletin no. 2 provided by Applied Biosystems.

### Western Blotting

Equal amounts of TriReagent® extracted proteins (20 µg) were separated electrically on 10% SDS–PAGE gel electrophoresis. The gels were electrophoresed, followed by a transfer of the protein to a nitrocellulose membrane. The membrane was then blocked with a blocking solution and probed with primary antibodies overnight at 4°C. The primary antibodies and concentrations used in western blot analysis were as follows: CxCR4 (1∶500, ab2074, Abcam, Cambridge, UK), SOCS2 (1∶100, ab3692, Abcam, Cambridge, UK), and TNFAIP3 (1∶200, sc-8432, Santa Cruz Biotechnology Inc., Santa Cruz, CA, USA). Immunoblots were next processed with appropriate secondary antibodies (either 1∶10000, HRP/goat anti-rabbit IgG, sc-2004, or 1∶10000 HRP/goat anti-mouse IgG, sc-2005, both from Santa Cruz Biotechnology Inc., Santa Cruz, CA, USA, based on the primary antibody) for 1 hour at room temperature. Bands were detected with a chemiluminescence reagent kit (Western lighting™ Chemiluminescence Reagent Plus, NEL104, Perkin Elmer LAS Inc., Boston, MA, USA). Blot bands were quantified by densitometry with Image J software (Image J 1.33u, NIH). β-Actin (1∶1000, sc-8435, Santa Cruz Biotechnology Inc., Santa Cruz, CA, USA) was blotted on the same membrane as a loading control for the protein extract. The data were expressed as relative densitometric units after background subtraction.

### Study Flow-Chart

Since our focus was understanding changes in transcripts associated to MS and modified during pregnancy, we devised a six steps analysis based on both microarray and real time RT-PCR procedures: 1) identification of transcripts associated to MS; 2) identification of transcripts losing their association to MS in pregnant patients; 3) validation of the transcripts by real time RT-PCR; 4) longitudinal analysis during gestation; 5) comparison of expression profiles of MS patients relapsing during pregnancy with those of relapse-free patients; 6) confirmation of the gene expression results on the protein level.

### Statistical Analysis

#### Array analysis

GeneChip Operating Software (GCOS) (Affymetrix, Santa Clara, CA) was used to generate background-normalized image data. Microarray quality controls and statistical validation was done using Bioconductor (www.bioconductor.org) [18]. The presence of hybridization/construction artifacts was evaluated with the fitPLM function (Bioconductor package affyPLM), and the probe (PM) intensity distribution was evaluated using hist function. Probe set intensities were obtained by means of RMA (Robust Multichip Average) and normalization was done according to the quantiles method [19], [20]. Probe sets from all experimental groups were filtered to have an inter-quartile range for each probe set >0.25 [21]. Subsequently, an intensity filter, removing all the probe sets characterized by a signal >6.64 in less than 10% of the analyzed arrays, was applied.

Feature selection of transcripts differentially expressed between MS and healthy specimens was addressed using the so-called rank product non-parametric method [22]. This method addresses the multiple comparison problem and performs *p*-value correction by false-discovery-rate (FDR), comparing the true rank product distribution with a random one defined permutating gene labels in each of the arrays under analysis. Here, we have used 500 permutations and a threshold of percentage of false positive predictions (pfp) of 0.05. Furthermore, specific pregnancy signatures were defined for both healthy controls and MS patients by mean of rank product analysis.

The PAMR method was subsequently used to refine the MS signature [23]. The subset derived from PAMR analysis was selected as the best compromise in transcripts number reduction and low error rate in Leave One-Out Cross-Validation (LOOCV). PAMR was also used to evaluate the efficacy of the MS signature during pregnancy, comparing MS and healthy specimens at ninth month pregnancy.

Parmigiani has devised an integrative correlation coefficient (ICC) for quantification of the extent to which independent studies can be reliably analyzed together for the systematic comparison of microarray profiles [24]. ICC ranges between zero and one; values closer to one indicate a strong correlation between the two groups. Here, this approach was used to detect what transcripts most differ among experimental conditions, i.e. MS patients *vs.* healthy controls before pregnancy, and MS patients *vs*. healthy controls at ninth month pregnancy. Specifically, all pair-wise correlations (Pearson coefficient) of gene expression across samples within MS *vs.* healthy conditions were calculated and the reproducibility of the results was defined without relying on direct comparison of expression across groups.

Microarray data were deposited on GEO database (www.ncbi.nlm.nih.gov/geo/) as superserie file (GSE17449), encompassing MS signature, MS pregnancy signature and healthy pregnancy signature.

Gene annotation and functional analysis was done with IPA 5.0 software (www.ingenuity.com).

#### Real time RT-PCR analysis

data were analyzed by parametric statistical tests, using GraphPad PRISM 4.00 (GraphPad Software, San Diego, CA). Expression levels of selected genes, according to categorical differences of both disease and pregnancy status were compared using the unpaired t-test, whereas comparisons of transcript levels at different time points were performed using the paired t-test. Two-way ANOVA was used to examine the effects of both disease and pregnancy on transcripts. All *p*-values were based on two-tailed statistical tests, with a significance level of 0.05.

## Results

### GeneChip Array

#### MS signature

**Figure 1:**
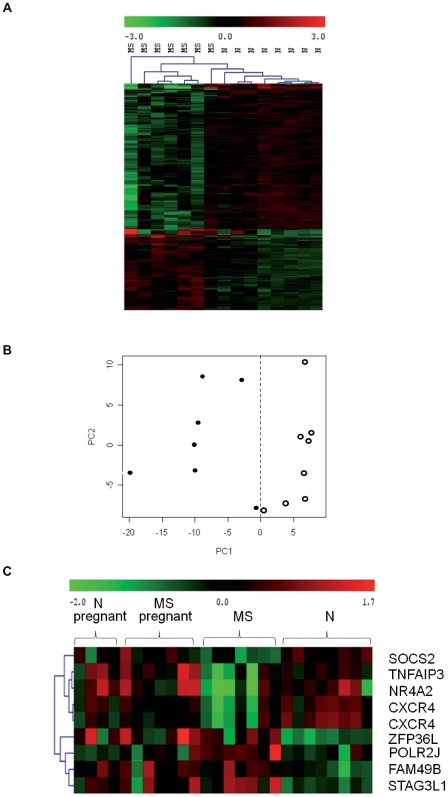
Microarray analysis of blood mRNAs from MS patients and normal controls before pregnancy. (A) Supervised clustering of the 347 most discriminating transcripts (i.e. 404 probe sets), that were defined as “MS signature”. Up-regulated genes are indicated by a red color, down-regulated by a green color and genes that show no differences in expression are indicated in black. Patients are indicated as MS, while healthy controls are indicated as N. (B) Principle Components Analysis (PCA) of the probe sets discriminating between 7 MS patients (close circles) and 9 healthy controls (i.e. 5 women who thereafter became pregnant +4 women who did not become pregnant) (open circles) before pregnancy. (C) Microarray heat map plot depicting expression changes of the set of eight transcripts, within the “MS signature”, whose expression was found to change most significantly during pregnancy.

Feature selection of transcripts characterizing MS was done comparing transcription profiles of MS patients *vs.* healthy controls, before pregnancy. A total of 404 transcripts was shown to be differentially expressed in patients as compared to healthy controls (pfp≤0.05) (Figure 1A, Table S1). Of these, 347 transcripts were considered the most discriminant gene-set by PAMR analysis (Table 3) [23].

**Table 3:** Cross-validation of the MS signature by the PAMR (Prediction Analysis for Microarrays) method in A) non-pregnant women and B) 9 months pregnant women.

*PAMR cross classification results on non-pregnant women.*	MS patients	Normal controls	Class Error rate
**MS patients**	7	0	0%
**Normal controls**	0	8	0%
			Overall error rate = 0.0

*PAMR cross classification results on 9 months pregnant women.*	MS patients	Normal controls	Class Error rate
**MS patients**	4	3	43%
**Normal controls**	1	3	25%
			Overall error rate = 0.34

Principle components analysis (PCA) on the pre-selected genes revealed a clear separation between healthy donors and MS patients (Figure 1B).

#### Changes in the MS signature occurring during pregnancy

Upon entering the second step of the analysis, we evaluated what changes in expression are detected in the MS signature, when nine months pregnant MS patients are compared with nine months pregnant healthy controls. Interestingly, during gestation the MS signature lost its discrimination power (Table 2). This agreed with the normalization of the MS phenotype during pregnancy. However, the event might be due to an experimental statistical power issue, and thus a validation on a larger cohort of patients was needed. On this ground, we have detected the transcripts displaying the most pronounced discrimination changes during pregnancy and subsequently performed an extended real time RT-PCR validation on these transcripts.

An arbitrary ICC threshold of 0.4 was used to detect transcripts that did not respond in a similar way in the two experimental conditions and therefore were associated with not only pregnancy, but also MS disease [24]. This analysis identified eight transcripts that displayed the most pronounced change during pregnancy as defined by an ICC<0.4. The eight transcripts included suppressor of cytokine signaling-2 (SOCS2), tumor necrosis factor alpha-induced protein-3 (TNFAIP3), nuclear receptor subfamily-4 member-2 (NR4A2), CXC chemokine receptor-4 (CxCR4), zinc-finger protein-36 C3H type-like-1 (ZFP36L1), polymerase (RNA) II polypeptide J (POLR2J), family with sequence similarity-49 member-B (FAM49B), and the stromal antigen-3 like-1 (STAG3L1).

### Real Time RT-PCR Data

#### Validation of selected transcripts

**Figure 2:**
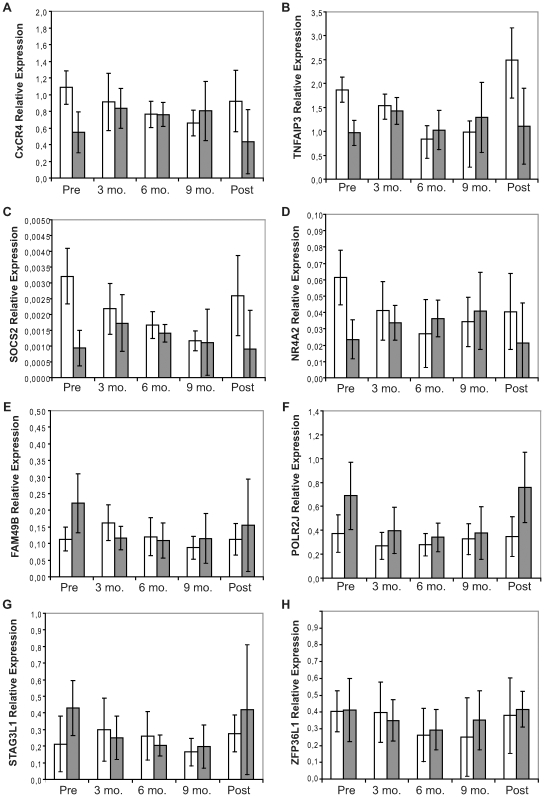
Average gene expression profiles in the overall cohort of MS patients and healthy controls. Comparison of average profiles of (A) CxCR4, (B) TNFAIP3, (C) SOCS2, (D) NR4A2, (E) FAM49B, (F) POLR2J, (G) STAG3L1, and (H) ZFP36L1 gene expressions in peripheral blood mononuclear cells obtained from 16 healthy controls (open columns) and 32 patients with multiple sclerosis (filled columns), followed during their pregnancy. Seven expression profiles from real time RT-PCR showed trends that agreed with the previous microarray profiles (A-G), while a single PCR results (i.e. ZFP36L1) (H) did not agree with that indicated by microarray: this gene showed no pregnancy-related regulation. Mean and bidirectional standard deviation values are indicated. The influence on the gene expressions of the time span between post-partum sample collection and delivery is comparable with the higher standard deviations observed for the latter time point.

Genes of interest were measured and directly validated by quantitative real time RT-PCR using the same patients and healthy controls' samples as tested by microarray. A further validation of the expression profiles was performed by using blood samples from an independent group of both MS patients and healthy controls followed during their pregnancy. In both validation processes, real time RT-PCR profiles agreed with microarray profiles for seven of the eight transcripts. Particularly, before pregnancy TNFAIP3, NR4A2, SOCS2, and CxCR4 were down-regulated in MS patients respect to healthy controls (all *p*≤0.0001). On the contrary, POLR2J, STAG3L1, and FAM49B were up-regulated in MS patients with respect to healthy controls (all *p*≤0.0033) (Figure 2).

#### Longitudinal analysis

As shown by longitudinal analysis, the overall deregulation of gene expression reverted to “normal” during gestation; specifically, pregnancy was shown to revert the imbalance already within the third month of gestation (all *p* = NS). On the contrary, in the post-partum gene expressions rebounded to levels which were significantly different between MS patients and healthy controls (all *p*≤0.01) and similar to the pre-pregnancy levels (Figure 2A–G). Clearly, since the time span of collection of the post-partum samples stretches over several months, corresponding results should be viewed carefully and variability should be taken into account.

Considering both validation processes, PCR results for ZFP36L1 did not agree with those indicated by microarray, as this latter transcript did not show differences in expression before pregnancy, and no pregnancy-related regulation was evident (Figure 2H).

Two-way ANOVA was used to determine the effects of MS disease and pregnancy for gene expression levels. The idea is that both MS disease and pregnancy might affect the gene expression.

Results showed that the individual effect on gene expression of both pregnancy and MS disease could not be measured for CxCR4, TNFAIP3, NR4A2, and FAM49B, even though parameter effect was observed on their own (all *p*≥0.080). For these genes it was clear that both disease and pregnancy have a different effect at different time points: when the progress of pregnancy is decreasing gene expression in healthy controls, the disease status increases that same gene expression, at the same time point. Therefore both effects annihilated each other, but an interaction between pregnancy and disease status was found to be significant (*p*≤0.021). Differently, pregnancy had a large effect on SOCS2 and STAG3L1 gene expression (both *p*≤0.0158), but not the disease status (both *p*≥0.1540). However, since pregnancy seemed to have different effect between healthy controls and MS patients, we concluded that there was interaction between disease and pregnancy.

Finally, two-way ANOVA revealed a significant effect of both pregnancy (*p*≤0.0001) and MS disease (*p*≤0.0001) with no significant interaction (*p* = 0.060) on the expression of POLJ2R.

#### Clinical analysis

**Figure 3:**
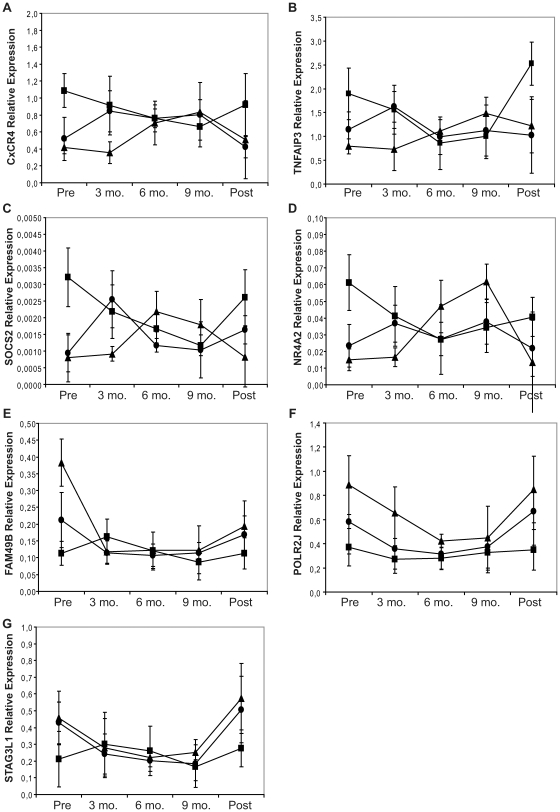
Average gene expressions in the overall cohorts of healthy controls and MS patients, subdivided based on their clinical outcome. Real time RT-PCR comparison of the average profiles of (A) CxCR4, (B) TNFAIP3, (C) SOCS2, (D) NR4A2, (E) FAM49B, (F) POLR2J, and (G) STAG3L1 gene expressions in peripheral blood mononuclear cells, obtained from 16 healthy controls and 32 patients with multiple sclerosis. MS patients were subdivided based on their clinical outcome, i.e. expression profiles of MS patients relapsing during pregnancy (triangles, n = 6) were compared with those of relapse-free patients (circles, n = 26) and healthy controls (squares, n = 16). Mean and bidirectional standard deviation values are indicated.

To explore whether the seven validated genes have any clinical relevance, we compared the expression profiles of MS patients relapsing during pregnancy with those of relapse-free patients. Six (18.7%) of the overall 32 MS patients had a relapse during pregnancy, prevailing in the first trimester; a single patients relapsed twice, in the first and in the third trimester respectively. Interestingly, in relapse-free patients the imbalance was reverted within the third month of gestation, whereas in patients who had a relapse during pregnancy, the gene expression imbalance was reverted later during gestation, i.e. at sixth month. Particularly, delayed reversal of expression profiles was observed for CxCR4, TNFAIP3, SOCS2, NR4A2, and POLR2J (Figure 3).

Two-way ANOVA showed that pregnancy had a large effect on both FAM49B and STAG3L1 expressions (% of total variation ≥16.45, both *p*≤0.0002), but not the relapse (% of total variation ≤1.50, both *p*≥0.168). On the contrary, on TNFAIP3 and CxCR4 expression, relapse had a quite significant effect (% of total variation ≥3.30, *p*≤0.050), but not pregnancy (% of total variation ≤1.57, both *p*≥0.6). Finally, both relapse and pregnancy showed a similar effect on POLJ2R transcript (% of total variation  = 7.42 and 21.65, respectively; both *p*≤0.0007), without interaction.

Individual effect on gene expression of both pregnancy and relapse could not be measured for NR4A2 and SOCS2 (all *p*≥0.172).

### Protein Expression

**Figure 4:**
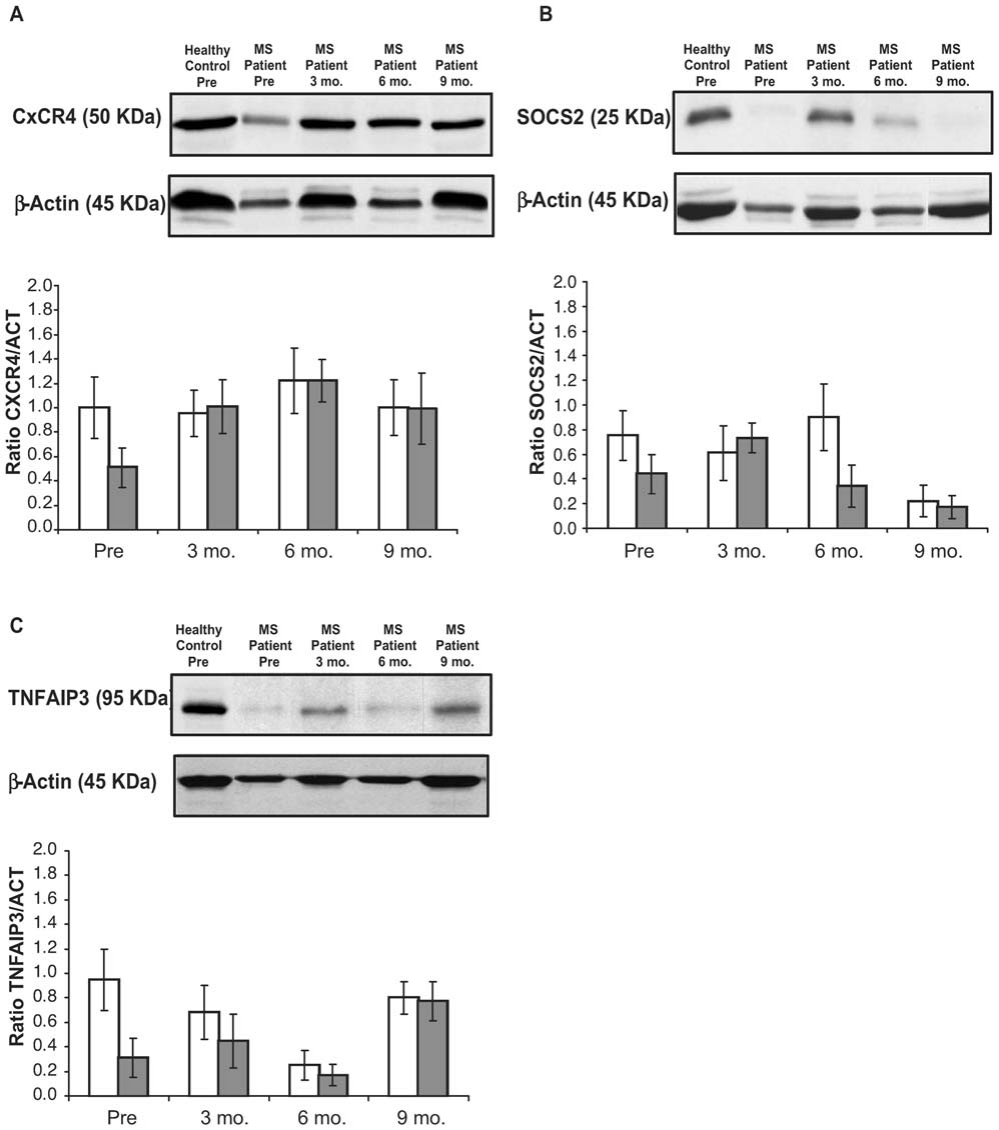
Protein expression of the CxCR4, SOCS2, and TNFAIP3 genes. Representative western blot bands of (A) CxCR4, (B) SOCS2, and (C) TNFAIP3 in PBMCs of a healthy control (blood sample taken before pregnancy) and a MS patient (blood samples taken before and during pregnancy). Corresponding β-Actin bands as loading control are shown in the upper panels. The lower panels in A–C show quantification of the western blot analysis for the respective protein. Particularly, the panels show the comparison of average profiles (with standard deviations) of (A) CxCR4, (B) SOCS2, (C) TNFAIP3 protein expressions in PBMCs obtained from 3 healthy controls (open columns) and 6 patients with multiple sclerosis (filled columns), followed during their pregnancy.

In order to confirm the gene expression results on the protein level, we compared the mRNA levels to CxCR4, SOCS2 and TNFAIP3 protein expression measured by western blot on the same samples obtained from 6 MS patients and 3 healthy controls, followed during their pregnancy. For the three genes, significant correlations were found between mRNA results and protein amounts. Particularly, we found that, similar to mRNA transcripts, before pregnancy TNFAIP3, SOCS2, and CxCR4 protein level were down-regulated in MS patients respect to healthy controls (all *p*≤0.004), while the overall deregulation of protein expression reverted to “normal” during gestation (Figure 4).

## Discussion

In order to identify modulated gene expression patterns, which correlate with the beneficial effect of pregnancy on disease course in MS, we undertook a genomic approach in profiling the genes expressed in PBMCs of MS patients and healthy volunteers, before, during, and after pregnancy.

First, a gene expression-based signature of MS was defined using genome-wide gene expression profiling. Of the 14564 genes on the array, 347 genes were found to have significantly altered expression in non-pregnant MS patients with respect to non-pregnant healthy controls. Since the MS phenotype seems to revert to a normal phenotype during pregnancy, we have then evaluated what changes in expression are detected in the MS signature, when nine months pregnant MS patients are compared with nine months pregnant healthy controls. Interestingly, pregnancy resulted in the reversion of the MS-related expression profile, as during pregnancy the MS signature lost its discrimination power. Longitudinal analysis showed that the overall deregulation of gene expression reverted to “normal” already within the third month of gestation, while during the post-partum gene expressions rebounded to levels which were similar to the pre-pregnancy levels and thus significantly different between MS patients and healthy controls.

Eight transcripts, within the initial MS signature, displayed the most pronounced change during pregnancy. These transcripts included SOCS2, TNFAIP3, NR4A2, CxCR4, ZFP36L1, POLR2J, FAM49B, and STAG3L1. Data were also corroborated by quantitative real time RT-PCR, showing discordance for only one transcript, i.e. ZFP36L1. For this transcript, microarray results indicated a pregnancy-related regulation, while real time RT-PCR revealed no changes in expression across the experimental conditions. Interestingly, all of the validated transcripts, with the exception of FAM49B and STAG3L1, which are genes encoding proteins of as yet unknown function, have been previously reported to be associated with MS and other autoimmune diseases [25]–[31]. These transcripts indeed, regulate several important inflammatory processes: TNFAIP3 is critical in limiting inflammation by terminating TNF-induced NFκB responses [32], NR4A2 has an important role in mediating multiple inflammatory signals [30], [33], [34], SOCS2 inhibits signal transduction induced by several cytokines including IL-6 [29], [35], CxCR4 plays a crucial role in the trafficking of T_reg_ cells [36], [37], and POLR2J is deregulated in some inflammatory diseases [25]. The involvement of inflammation-related genes in both pregnancy and MS outcome seems likely, as a regulation of inflammation is supportive for successful reproduction, and also positively influences MS [9]–[13]. Notably, we found expression profiles which were in line with the inflammatory nature of MS in non-pregnant MS patients, as well as with the anti-inflammatory milieu observed during pregnancy. Moreover, it is noteworthy that most of these transcripts have been reported to be regulated by estrogenic hormones [38]–[40], thus strengthening the main immunological mechanisms of adaptation of the maternal immune system during pregnancy.

Another interesting feature of our data derives from the observation that gene expression profiles correlated with the clinical outcome in MS patients. Indeed, MS patients relapsing during pregnancy showed different expression profiles when compared to relapse-free patients, as they displayed delayed reversal of expression, namely at month six or nine, compared to three months in relapse-free patients. This was particularly evident for the five above mentioned inflammation-related genes.

While on one side the present study shows an early pregnancy-induced up-regulation of the CxCR4, TNFAIP3, SOCS2, and NR4A2 transcripts in MS patients, on the other side data reveal a pregnancy-related down-regulation of the same genes, in healthy controls, as well as in MS patients after the achievement of a healthy gene expression profile. These reductions in expression observed during gestation correlate with the respective role of the genes in pregnancy: SOCS2 negatively regulates growth hormone action and inhibits prolactin signal transduction [41], thus its down-regulation is important for a successful pregnancy. Likewise, TNFAIP3 is involved in trophoblast invasion and its expression is physiologic in early pregnancy, but its up-regulation in later pregnancy augurs poorly [42]. Regarding CxCR4, a similar down-regulation was seen in the early placental tissue as compared to the term from healthy women [43]. This late down-regulation strongly suggests that CxCR4 has a role in the earlier part of pregnancy, when embryogenesis and much of the organogenesis takes place.

As regarding the NR4A2 transcript, at first glance its expression contradicts earlier works where NR4A2 was shown to be up-regulated in MS patients with respect to healthy controls [28], [29]. However, the difference may be explicable in terms of different experimental conditions: in the previous study, expression profiling was performed in specific cell type (i.e. T cells), whereas PBMCs, as used here, include various cell subtypes. Furthermore, the present study analyzed patients with no active MS before enrolment, while previous work included clinically different patients [44]. On the other hand, recent studies demonstrated a potent anti-inflammatory activity of NR4A2 in microglia and astrocytes, showing that quantitative defects in the expression or activities of this protein could predispose certain organ systems to inflammation-sensitive pathologies [33]–[34].

Given the present experimental design, it is possible that some of the changes in expression noted reflect a shift in the relative distributions of different subpopulations rather than real changes in gene expression. Although this is unlikely to be the case for molecules such as CxCR4, SOCS2, TNFAIP3 which are almost universally expressed by blood cells in response to inflammation, it could account for some of the apparent changes in expression of genes known to be highly expressed by specific cell types, e.g. NR4A2, which is mainly expressed by myeloid cells [45]. Nevertheless, although further studies on PBMCs subsets are logically needed, it is notable that presented changes are associated with a decrease in disease activity assessed by the occurrence of relapses during pregnancy. Hence, although limited by the low number of subjects, this is the first study in which the pregnancy-related decrease in disease activity of MS is investigated at a transcriptional level, a finding that broads our understanding of the pathogenesis of MS and might prove useful for the development of new therapeutic strategies.

## Supporting Information

Table S1 List of genes of transcripts characterizing MS.
(0.71 MB DOC)
