# Supplementary material for: Learning from Nature: Pregnancy Changes the Expression of Inflammation-Related Genes in Patients with Multiple Sclerosis
Source: PLoS One. 2010 Jan 29;5(1):e8962. doi: 10.1371/journal.pone.0008962 (PMC2813302; doi:10.1371/journal.pone.0008962)
Supplement: Table S1 — List of genes of transcripts characterizing MS. (0.71 MB DOC) [file pone.0008962.s001.doc]

**Table S-1**: list of genes of transcripts characterizing MS. Feature selection yielded 404 differentially expressed transcripts in MS patients compared to healthy controls, before pregnancy. Of these, 347 transcripts were considered the most discriminant gene-set by PAMR analysis.

| **AffyID** | **EG** | **Symbol** | **MS**  **score** | **N**  **score** |
| --- | --- | --- | --- | --- |
| 200672_x_at | 6711 | SPTBN1 | -0.4858 | 0.3778 |
| 200678_x_at | 2896 | GRN | 0.7649 | -0.5949 |
| 200824_at | 2950 | GSTP1 | 0.6875 | -0.5348 |
| 200923_at | 3959 | LGALS3BP | 0.6806 | -0.5293 |
| 201050_at | 23646 | PLD3 | 0.6722 | -0.5228 |
| 201066_at | 1537 | CYC1 | 0.7592 | -0.5905 |
| 201083_s_at | 9774 | BCLAF1 | -0.4807 | 0.3739 |
| 201101_s_at | 9774 | BCLAF1 | -0.4841 | 0.3765 |
| 201151_s_at | 4154 | MBNL1 | -0.666 | 0.518 |
| 201211_s_at | 1654 | DDX3X | -0.6118 | 0.4758 |
| 201237_at | 830 | CAPZA2 | -0.4637 | 0.3607 |
| 201281_at | 11047 | ADRM1 | 0.6605 | -0.5137 |
| 201284_s_at | 327 | APEH | 0.6125 | -0.4764 |
| 201307_at | 55752 | SEPT11 | -0.5066 | 0.394 |
| 201450_s_at | 7072 | TIA1 | -0.6094 | 0.474 |
| 201604_s_at | 4659 | PPP1R12A | -0.6916 | 0.5379 |
| 201663_s_at | 10051 | SMC4 | -0.491 | 0.3819 |
| 201689_s_at | 7163 | TPD52 | -0.5935 | 0.4616 |
| 201693_s_at | 1958 | EGR1 | 0.4582 | -0.3563 |
| 201734_at | 1182 | CLCN3 | -0.5333 | 0.4148 |
| 201770_at | 6626 | SNRPA | 0.797 | -0.6199 |
| 201850_at | 822 | CAPG | 0.7251 | -0.564 |
| 202009_at | 11344 | TWF2 | 0.7105 | -0.5526 |
| 202034_x_at | 9821 | RB1CC1 | -0.5498 | 0.4276 |
| 202073_at | 10133 | OPTN | -0.7623 | 0.5929 |
| 202097_at | 9972 | NUP153 | -0.5725 | 0.4453 |
| 202145_at | 4061 | LY6E | 0.5865 | -0.4562 |
| 202161_at | 5585 | PKN1 | 0.5883 | -0.4576 |
| 202180_s_at | 9961 | MVP | 0.6576 | -0.5115 |
| 202201_at | 645 | BLVRB | 0.5351 | -0.4162 |
| 202275_at | 2539 | G6PD | 0.6995 | -0.5441 |
| 202307_s_at | 6890 | TAP1 | 0.6203 | -0.4824 |
| 202318_s_at | 26054 | SENP6 | -0.619 | 0.4815 |
| 202380_s_at | 4820 | NKTR | -0.5805 | 0.4515 |
| 202426_s_at | 6256 | RXRA | 0.5894 | -0.4584 |
| 202457_s_at | 5530 | PPP3CA | -0.5793 | 0.4505 |
| 202498_s_at | 6515 | SLC2A3 | -0.5358 | 0.4167 |
| 202535_at | 8772 | FADD | 0.5275 | -0.4103 |
| 202545_at | 5580 | PRKCD | 0.5814 | -0.4522 |
| 202599_s_at | 8204 | NRIP1 | -0.5639 | 0.4386 |
| 202630_at | 10513 | APPBP2 | -0.4798 | 0.3732 |
| 202643_s_at | 7128 | TNFAIP3 | -0.4506 | 0.3505 |
| 202660_at | 3709 | ITPR2 | -0.5695 | 0.4429 |
| 202671_s_at | 8566 | PDXK | 0.7658 | -0.5956 |
| 202730_s_at | 27250 | PDCD4 | -0.5072 | 0.3945 |
| 202731_at | 27250 | PDCD4 | -0.5707 | 0.4439 |
| 202742_s_at | 5567 | PRKACB | -0.5931 | 0.4613 |
| 202759_s_at | 11217 | AKAP2 | -0.5376 | 0.4182 |
| 202761_s_at | 23224 | SYNE2 | -0.6134 | 0.4771 |
| 202762_at | 9475 | ROCK2 | -0.6673 | 0.519 |
| 202767_at | 53 | ACP2 | 0.5856 | -0.4555 |
| 202787_s_at | 7867 | MAPKAPK3 | 0.6151 | -0.4784 |
| 202814_s_at | 10614 | HEXIM1 | -0.6238 | 0.4851 |
| 202817_s_at | 6760 | SS18 | -0.5305 | 0.4126 |
| 202842_s_at | 4189 | DNAJB9 | -0.6051 | 0.4707 |
| 202843_at | 4189 | DNAJB9 | -0.5762 | 0.4481 |
| 202855_s_at | 9123 | SLC16A3 | 0.455 | -0.3539 |
| 202856_s_at | 9123 | SLC16A3 | 0.5038 | -0.3918 |
| 202859_x_at | 3576 | IL8 | -0.5998 | 0.4665 |
| 202947_s_at | 2995 | GYPC | 0.5283 | -0.4109 |
| 202979_s_at | 58487 | CREBZF | -0.5001 | 0.389 |
| 202988_s_at | 5996 | RGS1 | -0.5405 | 0.4204 |
| 203045_at | 4814 | NINJ1 | 0.5382 | -0.4186 |
| 203049_s_at | 9652 | TTC37 | -0.6498 | 0.5054 |
| 203080_s_at | 29994 | BAZ2B | -0.6055 | 0.471 |
| 203156_at | 11215 | AKAP11 | -0.6078 | 0.4728 |
| 203167_at | 7077 | TIMP2 | 0.5218 | -0.4059 |
| 203175_at | 391 | RHOG | 0.6798 | -0.5287 |
| 203236_s_at | 3965 | LGALS9 | 0.7494 | -0.5829 |
| 203248_at | 7572 | ZNF24 | -0.589 | 0.4581 |
| 203310_at | 6814 | STXBP3 | -0.5403 | 0.4203 |
| 203373_at | 8835 | SOCS2 | -0.6602 | 0.5135 |
| 203387_s_at | 9882 | TBC1D4 | -0.5742 | 0.4466 |
| 203519_s_at | 26019 | UPF2 | -0.6437 | 0.5006 |
| 203525_s_at | 324 | APC | -0.687 | 0.5343 |
| 203543_s_at | 687 | KLF9 | -0.6069 | 0.4721 |
| 203553_s_at | 11183 | MAP4K5 | -0.6499 | 0.5055 |
| 203566_s_at | 178 | AGL | -0.5711 | 0.4442 |
| 203593_at | 23607 | CD2AP | -0.608 | 0.4729 |
| 203603_s_at | 9839 | ZEB2 | -0.6918 | 0.5381 |
| 203640_at | 10150 | MBNL2 | -0.5819 | 0.4526 |
| 203652_at | 4296 | MAP3K11 | 0.8539 | -0.6642 |
| 203708_at | 5142 | PDE4B | -0.4943 | 0.3844 |
| 203719_at | 2067 | ERCC1 | 0.6757 | -0.5255 |
| 203791_at | 1657 | DMXL1 | -0.5323 | 0.414 |
| 203845_at | 8850 | KAT2B | -0.6681 | 0.5197 |
| 203973_s_at | 1052 | CEBPD | 0.6251 | -0.4862 |
| 203989_x_at | 2149 | F2R | -0.6411 | 0.4987 |
| 204018_x_at | 3039 | HBA1 | 0.5058 | -0.3934 |
| 204039_at | 1050 | CEBPA | 0.7779 | -0.605 |
| 204049_s_at | 9749 | PHACTR2 | -0.6705 | 0.5215 |
| 204071_s_at | 10210 | TOPORS | -0.533 | 0.4146 |
| 204099_at | 6604 | SMARCD3 | 0.5866 | -0.4563 |
| 204160_s_at | 22875 | ENPP4 | -0.664 | 0.5165 |
| 204286_s_at | 5366 | PMAIP1 | -0.5223 | 0.4062 |
| 204299_at | 10772 | FUSIP1 | -0.7081 | 0.5507 |
| 204314_s_at | 1385 | CREB1 | -0.5667 | 0.4408 |
| 204419_x_at | 3048 | HBG2 | 0.4912 | -0.382 |
| 204480_s_at | 79095 | C9orf16 | 0.5478 | -0.4261 |
| 204491_at | 5144 | PDE4D | -0.4964 | 0.3861 |
| 204494_s_at | 56905 | C15orf39 | 0.6928 | -0.5389 |
| 204512_at | 3096 | HIVEP1 | -0.4744 | 0.369 |
| 204610_s_at | 11007 | CCDC85B | 0.6914 | -0.5377 |
| 204621_s_at | 4929 | NR4A2 | -0.543 | 0.4223 |
| 204622_x_at | 4929 | NR4A2 | -0.6854 | 0.5331 |
| 204638_at | 54 | ACP5 | 0.4949 | -0.3849 |
| 204645_at | 905 | CCNT2 | -0.5441 | 0.4232 |
| 204731_at | 7049 | TGFBR3 | -0.5814 | 0.4522 |
| 204848_x_at | 3047 | HBG1 | 0.4728 | -0.3678 |
| 204858_s_at | 1890 | TYMP | 0.641 | -0.4986 |
| 204897_at | 5734 | PTGER4 | -0.5882 | 0.4575 |
| 204917_s_at | 4300 | MLLT3 | -0.6185 | 0.4811 |
| 205005_s_at | 9397 | NMT2 | -0.5345 | 0.4157 |
| 205006_s_at | 9397 | NMT2 | -0.6179 | 0.4806 |
| 205027_s_at | 1326 | MAP3K8 | -0.5715 | 0.4445 |
| 205091_x_at | 5965 | RECQL | -0.5233 | 0.407 |
| 205147_x_at | 4689 | NCF4 | 0.5613 | -0.4366 |
| 205237_at | 2219 | FCN1 | 0.6436 | -0.5006 |
| 205259_at | 4306 | NR3C2 | -0.6146 | 0.478 |
| 205321_at | 1968 | EIF2S3 | -0.5711 | 0.4442 |
| 205349_at | 2769 | GNA15 | 0.7508 | -0.584 |
| 205418_at | 2242 | FES | 0.5336 | -0.415 |
| 205419_at | 1880 | GPR183 | -0.5269 | 0.4098 |
| 205442_at | 9848 | MFAP3L | -0.4444 | 0.3457 |
| 205469_s_at | 3663 | IRF5 | 0.7036 | -0.5473 |
| 205483_s_at | 9636 | ISG15 | 0.5305 | -0.4126 |
| 205518_s_at | 8418 | CMAH | -0.6324 | 0.4918 |
| 205668_at | 4065 | LY75 | -0.6717 | 0.5224 |
| 205819_at | 8685 | MARCO | 0.7499 | -0.5833 |
| 205875_s_at | 11277 | TREX1 | 0.7996 | -0.6219 |
| 205884_at | 3676 | ITGA4 | -0.4514 | 0.3511 |
| 205936_s_at | 3101 | HK3 | 0.6093 | -0.4739 |
| 206003_at | 9662 | CEP135 | -0.6274 | 0.4879 |
| 206074_s_at | 3159 | HMGA1 | 0.7627 | -0.5932 |
| 206108_s_at | 6431 | SFRS6 | -0.7098 | 0.5521 |
| 206130_s_at | 433 | ASGR2 | 0.5255 | -0.4087 |
| 206207_at | 1178 | CLC | -0.8391 | 0.6526 |
| 206380_s_at | 5199 | CFP | 0.6538 | -0.5085 |
| 206545_at | 940 | CD28 | -0.5179 | 0.4028 |
| 206618_at | 8809 | IL18R1 | -0.5456 | 0.4244 |
| 206637_at | 9934 | P2RY14 | -0.5222 | 0.4061 |
| 206761_at | 10225 | CD96 | -0.6152 | 0.4785 |
| 206828_at | 7294 | TXK | -0.6252 | 0.4863 |
| 206875_s_at | 9748 | SLK | -0.5559 | 0.4323 |
| 207108_s_at | 25836 | NIPBL | -0.5044 | 0.3923 |
| 207358_x_at | 23499 | MACF1 | -0.6417 | 0.4991 |
| 207535_s_at | 4791 | NFKB2 | 0.6174 | -0.4802 |
| 207563_s_at | 8473 | OGT | -0.5946 | 0.4625 |
| 207564_x_at | 8473 | OGT | -0.6654 | 0.5176 |
| 207723_s_at | 3823 | KLRC3 | -0.4713 | 0.3665 |
| 207840_at | 11126 | CD160 | -0.5331 | 0.4146 |
| 207857_at | 11027 | LILRA2 | 0.6061 | -0.4714 |
| 208436_s_at | 3665 | IRF7 | 0.5365 | -0.4173 |
| 208498_s_at | 276 | AMY1A | -0.5163 | 0.4016 |
| 208798_x_at | 23015 | GOLGA8A | -0.7009 | 0.5452 |
| 208802_at | 6731 | SRP72 | -0.5053 | 0.393 |
| 208818_s_at | 1312 | COMT | 0.7401 | -0.5757 |
| 208861_s_at | 546 | ATRX | -0.6258 | 0.4867 |
| 208890_s_at | 23654 | PLXNB2 | 0.718 | -0.5584 |
| 208893_s_at | 1848 | DUSP6 | 0.5138 | -0.3996 |
| 208928_at | 5447 | POR | 0.6709 | -0.5218 |
| 208995_s_at | 9360 | PPIG | -0.5718 | 0.4447 |
| 209112_at | 1027 | CDKN1B | -0.5778 | 0.4494 |
| 209116_x_at | 3043 | HBB | 0.4485 | -0.3488 |
| 209166_s_at | 4125 | MAN2B1 | 0.6827 | -0.531 |
| 209179_s_at | 79143 | MBOAT7 | 0.7125 | -0.5542 |
| 209201_x_at | 7852 | CXCR4 | -0.5953 | 0.463 |
| 209259_s_at | 9126 | SMC3 | -0.4804 | 0.3737 |
| 209271_at | 2186 | BPTF | -0.7705 | 0.5993 |
| 209281_s_at | 490 | ATP2B1 | -0.5372 | 0.4178 |
| 209285_s_at | 23272 | C3orf63 | -0.5286 | 0.4111 |
| 209307_at | 23075 | SWAP70 | -0.744 | 0.5786 |
| 209348_s_at | 4094 | MAF | -0.6523 | 0.5073 |
| 209367_at | 6813 | STXBP2 | 0.625 | -0.4861 |
| 209379_s_at | 54462 | KIAA1128 | -0.6658 | 0.5179 |
| 209421_at | 4436 | MSH2 | -0.5663 | 0.4404 |
| 209422_at | 51230 | PHF20 | -0.5958 | 0.4634 |
| 209451_at | 10010 | TANK | -0.4962 | 0.3859 |
| 209458_x_at | 3039 | HBA1 | 0.4944 | -0.3845 |
| 209499_x_at | 8741 | TNFSF13 | 0.7497 | -0.5831 |
| 209500_x_at | 8741 | TNFSF13 | 0.6759 | -0.5257 |
| 209695_at | 11156 | PTP4A3 | 0.7137 | -0.5551 |
| 209704_at | 22823 | MTF2 | -0.5391 | 0.4193 |
| 209750_at | 9975 | NR1D2 | -0.7066 | 0.5496 |
| 209754_s_at | 7112 | TMPO | -0.4913 | 0.3821 |
| 209782_s_at | 1628 | DBP | 0.5901 | -0.459 |
| 209795_at | 969 | CD69 | -0.6281 | 0.4886 |
| 209815_at | 5727 | PTCH1 | -0.6159 | 0.479 |
| 209820_s_at | 10607 | TBL3 | 0.5896 | -0.4586 |
| 209829_at | 9750 | FAM65B | -0.6815 | 0.5301 |
| 209884_s_at | 9497 | SLC4A7 | -0.5249 | 0.4082 |
| 209930_s_at | 4778 | NFE2 | 0.5249 | -0.4083 |
| 209994_s_at | 5243 | ABCB1 | -0.6566 | 0.5107 |
| 210044_s_at | 4066 | LYL1 | 0.7083 | -0.5509 |
| 210077_s_at | 6430 | SFRS5 | -0.7147 | 0.5559 |
| 210148_at | 10114 | HIPK3 | -0.6749 | 0.5249 |
| 210162_s_at | 4772 | NFATC1 | 0.5895 | -0.4585 |
| 210205_at | 8705 | B3GALT4 | 0.5782 | -0.4497 |
| 210254_at | 932 | MS4A3 | -0.5169 | 0.402 |
| 210284_s_at | 23118 | MAP3K7IP2 | -0.672 | 0.5227 |
| 210314_x_at | 8741 | TNFSF13 | 0.7503 | -0.5836 |
| 210356_x_at | 931 | MS4A1 | -0.4556 | 0.3544 |
| 210424_s_at | 23015 | GOLGA8A | -0.8089 | 0.6291 |
| 210425_x_at | 23015 | GOLGA8A | -0.925 | 0.7195 |
| 210613_s_at | 9145 | SYNGR1 | 0.5947 | -0.4625 |
| 210621_s_at | 5921 | RASA1 | -0.6788 | 0.528 |
| 210676_x_at | 84220 | RGPD5 | -0.5586 | 0.4345 |
| 211090_s_at | 8899 | PRPF4B | -0.5671 | 0.4411 |
| 211100_x_at | 11027 | LILRA2 | 0.5893 | -0.4583 |
| 211101_x_at | 11027 | LILRA2 | 0.5856 | -0.4555 |
| 211102_s_at | 11027 | LILRA2 | 0.6362 | -0.4948 |
| 211121_s_at | 1796 | DOK1 | 0.6426 | -0.4998 |
| 211136_s_at | 1209 | CLPTM1 | 0.5415 | -0.4211 |
| 211284_s_at | 2896 | GRN | 0.7838 | -0.6096 |
| 211302_s_at | 5142 | PDE4B | -0.5122 | 0.3984 |
| 211506_s_at | 3576 | IL8 | -0.5954 | 0.4631 |
| 211576_s_at | 6573 | SLC19A1 | 0.8225 | -0.6397 |
| 211675_s_at | 29969 | MDFIC | -0.7206 | 0.5604 |
| 211699_x_at | 3039 | HBA1 | 0.5056 | -0.3932 |
| 211734_s_at | 2205 | FCER1A | -0.5569 | 0.4331 |
| 211745_x_at | 3039 | HBA1 | 0.4826 | -0.3754 |
| 211919_s_at | 7852 | CXCR4 | -0.6156 | 0.4788 |
| 211965_at | 677 | ZFP36L1 | 0.4753 | -0.3697 |
| 212030_at | 58517 | RBM25 | -0.5541 | 0.431 |
| 212090_at | 2907 | GRINA | 0.6 | -0.4667 |
| 212223_at | 3423 | IDS | -0.5568 | 0.4331 |
| 212239_at | 5295 | PIK3R1 | -0.4783 | 0.372 |
| 212286_at | 23253 | ANKRD12 | -0.6277 | 0.4882 |
| 212289_at | 23253 | ANKRD12 | -0.525 | 0.4084 |
| 212307_s_at | 8473 | OGT | -0.6349 | 0.4938 |
| 212373_at | 10116 | FEM1B | -0.5161 | 0.4014 |
| 212402_at | 23091 | ZC3H13 | -0.6383 | 0.4964 |
| 212412_at | 10611 | PDLIM5 | -0.639 | 0.497 |
| 212462_at | 23522 | MYST4 | -0.6815 | 0.5301 |
| 212512_s_at | 10498 | CARM1 | 0.6924 | -0.5386 |
| 212514_x_at | 1654 | DDX3X | -0.6148 | 0.4782 |
| 212515_s_at | 1654 | DDX3X | -0.5748 | 0.4471 |
| 212520_s_at | 6597 | SMARCA4 | 0.6439 | -0.5008 |
| 212538_at | 23348 | DOCK9 | -0.5517 | 0.4291 |
| 212563_at | 23246 | BOP1 | 0.5379 | -0.4184 |
| 212614_at | 84159 | ARID5B | -0.4633 | 0.3603 |
| 212628_at | 5586 | PKN2 | -0.54 | 0.42 |
| 212633_at | 23376 | KIAA0776 | -0.4864 | 0.3783 |
| 212641_at | 3097 | HIVEP2 | -0.4857 | 0.3778 |
| 212672_at | 472 | ATM | -0.6936 | 0.5395 |
| 212687_at | 3987 | LIMS1 | -0.5773 | 0.449 |
| 212704_at | 23318 | ZCCHC11 | -0.6819 | 0.5304 |
| 212720_at | 10914 | PAPOLA | -0.6793 | 0.5284 |
| 212721_at | 140890 | SFRS12 | -0.5402 | 0.4202 |
| 212764_at | 6935 | ZEB1 | -0.5938 | 0.4618 |
| 212779_at | 84162 | KIAA1109 | -0.6165 | 0.4795 |
| 212780_at | 6654 | SOS1 | -0.5394 | 0.4195 |
| 212847_at | 8880 | FUBP1 | -0.5359 | 0.4168 |
| 212867_at | NA | NA | -0.6585 | 0.5122 |
| 212930_at | 490 | ATP2B1 | -0.6699 | 0.521 |
| 212959_s_at | 79158 | GNPTAB | -0.6402 | 0.4979 |
| 213024_at | 7110 | TMF1 | -0.5366 | 0.4174 |
| 213025_at | 55623 | THUMPD1 | -0.515 | 0.4006 |
| 213049_at | 253959 | GARNL1 | -0.6643 | 0.5167 |
| 213070_at | 5286 | PIK3C2A | -0.5699 | 0.4433 |
| 213111_at | 200576 | PIP5K3 | -0.6409 | 0.4985 |
| 213156_at | NA | NA | -0.6094 | 0.4739 |
| 213158_at | NA | NA | -0.6283 | 0.4887 |
| 213165_at | 9857 | CEP350 | -0.5783 | 0.4498 |
| 213182_x_at | 1028 | CDKN1C | 0.4986 | -0.3878 |
| 213212_x_at | 374650 | FLJ40113 | -0.7353 | 0.5719 |
| 213225_at | 5495 | PPM1B | -0.4752 | 0.3696 |
| 213238_at | 57205 | ATP10D | -0.4732 | 0.368 |
| 213262_at | 26278 | SACS | -0.6092 | 0.4738 |
| 213311_s_at | 22980 | TCF25 | 0.6251 | -0.4862 |
| 213353_at | 23461 | ABCA5 | -0.6485 | 0.5044 |
| 213376_at | 22890 | ZBTB1 | -0.5749 | 0.4471 |
| 213405_at | 57403 | RAB22A | -0.473 | 0.3679 |
| 213470_s_at | 3187 | HNRNPH1 | -0.53 | 0.4123 |
| 213510_x_at | 220594 | LOC220594 | -0.7042 | 0.5477 |
| 213515_x_at | 3048 | HBG2 | 0.4995 | -0.3885 |
| 213549_at | NA | NA | -0.6051 | 0.4707 |
| 213716_s_at | 6398 | SECTM1 | 0.6941 | -0.5398 |
| 213906_at | 4603 | MYBL1 | -0.6989 | 0.5436 |
| 213918_s_at | 25836 | NIPBL | -0.599 | 0.4659 |
| 214012_at | 51752 | ERAP1 | -0.5435 | 0.4227 |
| 214124_x_at | NA | NA | -0.5672 | 0.4412 |
| 214132_at | 509 | ATP5C1 | -0.4827 | 0.3754 |
| 214290_s_at | 8337 | HIST2H2AA3 | 0.4644 | -0.3612 |
| 214414_x_at | 3039 | HBA1 | 0.47 | -0.3655 |
| 214470_at | 3820 | KLRB1 | -0.5393 | 0.4194 |
| 214499_s_at | 9774 | BCLAF1 | -0.5869 | 0.4565 |
| 214683_s_at | 1195 | CLK1 | -0.6282 | 0.4886 |
| 214697_s_at | 9991 | ROD1 | -0.5604 | 0.4358 |
| 214700_x_at | 55183 | RIF1 | -0.5091 | 0.396 |
| 214740_at | 246721 | POLR2J2 | 0.4491 | -0.3493 |
| 214746_s_at | 168544 | ZNF467 | 0.705 | -0.5483 |
| 214786_at | 4214 | MAP3K1 | -0.487 | 0.3788 |
| 214855_s_at | 253959 | GARNL1 | -0.6931 | 0.5391 |
| 214895_s_at | 102 | ADAM10 | -0.6714 | 0.5222 |
| 215009_s_at | 22872 | SEC31A | -0.4631 | 0.3602 |
| 215032_at | 6239 | RREB1 | 0.6115 | -0.4756 |
| 215137_at | 57244 | RP11-374F3.4 | 0.804 | -0.6254 |
| 215177_s_at | 3655 | ITGA6 | -0.5823 | 0.4529 |
| 215191_at | NA | NA | 0.5498 | -0.4276 |
| 215602_at | 221472 | FGD2 | 0.7374 | -0.5735 |
| 215716_s_at | 490 | ATP2B1 | -0.6932 | 0.5392 |
| 216041_x_at | 2896 | GRN | 0.7558 | -0.5878 |
| 216236_s_at | 144195 | SLC2A14 | -0.4871 | 0.3789 |
| 216248_s_at | 4929 | NR4A2 | -0.6997 | 0.5442 |
| 216563_at | 23253 | ANKRD12 | -0.5263 | 0.4093 |
| 216834_at | 5996 | RGS1 | -0.5653 | 0.4397 |
| 216835_s_at | 1796 | DOK1 | 0.6128 | -0.4767 |
| 217028_at | 7852 | CXCR4 | -0.4971 | 0.3867 |
| 217414_x_at | 3039 | HBA1 | 0.4957 | -0.3855 |
| 217418_x_at | 931 | MS4A1 | -0.4779 | 0.3717 |
| 217497_at | 1890 | TYMP | 0.7264 | -0.565 |
| 217503_at | 9262 | STK17B | -0.5175 | 0.4025 |
| 217534_at | 51571 | FAM49B | 0.5739 | -0.4464 |
| 217646_at | 6834 | SURF1 | 0.6253 | -0.4863 |
| 217691_x_at | 9123 | SLC16A3 | 0.4754 | -0.3698 |
| 217851_s_at | 51012 | SLMO2 | -0.548 | 0.4262 |
| 217977_at | 51734 | SEPX1 | 0.6252 | -0.4863 |
| 218040_at | 55119 | PRPF38B | -0.5609 | 0.4363 |
| 218145_at | 57761 | TRIB3 | 0.6321 | -0.4916 |
| 218197_s_at | 55074 | OXR1 | -0.4651 | 0.3618 |
| 218215_s_at | 7376 | NR1H2 | 0.7035 | -0.5471 |
| 218230_at | 27236 | ARFIP1 | -0.4543 | 0.3533 |
| 218247_s_at | 51320 | MEX3C | -0.5879 | 0.4572 |
| 218280_x_at | 8337 | HIST2H2AA3 | 0.4708 | -0.3662 |
| 218331_s_at | 54906 | C10orf18 | -0.6087 | 0.4734 |
| 218352_at | 55213 | RCBTB1 | -0.5356 | 0.4166 |
| 218387_s_at | 25796 | PGLS | 0.6729 | -0.5234 |
| 218458_at | 64395 | GMCL1 | -0.5899 | 0.4588 |
| 218603_at | 51696 | HECA | -0.6607 | 0.5139 |
| 218681_s_at | 23753 | SDF2L1 | 0.633 | -0.4923 |
| 218683_at | 58155 | PTBP2 | -0.659 | 0.5125 |
| 218711_s_at | 8436 | SDPR | -0.6143 | 0.4778 |
| 218713_at | 79664 | NARG2 | -0.4909 | 0.3818 |
| 218750_at | 79101 | TAF1D | -0.62 | 0.4822 |
| 218829_s_at | 55636 | CHD7 | -0.5847 | 0.4547 |
| 218842_at | 79657 | RPAP3 | -0.5065 | 0.3939 |
| 218872_at | 54997 | TESC | 0.7151 | -0.5562 |
| 218878_s_at | 23411 | SIRT1 | -0.6458 | 0.5023 |
| 218930_s_at | 54664 | TMEM106B | -0.4854 | 0.3775 |
| 219015_s_at | 79868 | ALG13 | -0.4635 | 0.3605 |
| 219049_at | 55790 | CSGALNACT1 | -0.5154 | 0.4009 |
| 219130_at | 54482 | CCDC76 | -0.5423 | 0.4218 |
| 219165_at | 64236 | PDLIM2 | 0.7336 | -0.5706 |
| 219256_s_at | 54436 | SH3TC1 | 0.7183 | -0.5587 |
| 219259_at | 64218 | SEMA4A | 0.6593 | -0.5128 |
| 219315_s_at | 79652 | TMEM204 | 0.4858 | -0.3779 |
| 219326_s_at | 10678 | B3GNT2 | -0.4755 | 0.3698 |
| 219342_at | 64921 | CASD1 | -0.4885 | 0.3799 |
| 219456_s_at | 79890 | RIN3 | 0.6633 | -0.5159 |
| 219540_at | 10308 | ZNF267 | -0.488 | 0.3796 |
| 219628_at | 64393 | ZMAT3 | -0.6189 | 0.4814 |
| 219673_at | 254394 | MCM9 | -0.5211 | 0.4053 |
| 219709_x_at | 65990 | FAM173A | 0.5447 | -0.4237 |
| 219797_at | 11320 | MGAT4A | -0.5012 | 0.3898 |
| 219806_s_at | 56935 | C11orf75 | 0.4711 | -0.3664 |
| 219848_s_at | 9668 | ZNF432 | -0.6117 | 0.4758 |
| 220012_at | 56605 | ERO1LB | -0.4857 | 0.3778 |
| 220038_at | 23678 | SGK3 | -0.5725 | 0.4453 |
| 220132_s_at | 29121 | CLEC2D | -0.5091 | 0.396 |
| 220162_s_at | 64170 | CARD9 | 0.6894 | -0.5362 |
| 220330_s_at | 64092 | SAMSN1 | -0.5274 | 0.4102 |
| 220369_at | 55671 | SMEK1 | -0.5884 | 0.4577 |
| 220386_s_at | 27436 | EML4 | -0.6061 | 0.4714 |
| 220532_s_at | 28959 | TMEM176B | 0.464 | -0.3609 |
| 220553_s_at | 55015 | PRPF39 | -0.5153 | 0.4008 |
| 220646_s_at | 51348 | KLRF1 | -0.7773 | 0.6046 |
| 220712_at | 619426 | C8orf60 | 0.49 | -0.3811 |
| 221020_s_at | 81034 | SLC25A32 | -0.6119 | 0.4759 |
| 221191_at | 54441 | STAG3L1 | 0.6574 | -0.5113 |
| 221211_s_at | 56911 | C21orf7 | -0.5027 | 0.391 |
| 221221_s_at | 26249 | KLHL3 | -0.5809 | 0.4518 |
| 221234_s_at | 60468 | BACH2 | -0.4823 | 0.3751 |
| 221428_s_at | 79718 | TBL1XR1 | -0.7161 | 0.5569 |
| 221545_x_at | 10025 | MED16 | 0.5551 | -0.4317 |
| 221556_at | 8555 | CDC14B | -0.4499 | 0.3499 |
| 221565_s_at | 51063 | CALHM2 | 0.511 | -0.3974 |
| 221638_s_at | 8675 | STX16 | -0.5319 | 0.4137 |
| 221645_s_at | 55769 | ZNF83 | -0.5041 | 0.3921 |
| 221666_s_at | 29108 | PYCARD | 0.8104 | -0.6303 |
| 221727_at | 10923 | SUB1 | -0.5003 | 0.3892 |
| 221751_at | 1E+08 | SLC2A3P1 | -0.6613 | 0.5143 |
| 221760_at | 4121 | MAN1A1 | -0.6009 | 0.4674 |
| 221763_at | 221037 | JMJD1C | -0.5234 | 0.4071 |
| 221768_at | 6421 | SFPQ | -0.6614 | 0.5144 |
| 221882_s_at | 58986 | TMEM8 | 0.6238 | -0.4852 |
| 221918_at | 5128 | PCTK2 | -0.547 | 0.4254 |
| 221919_at | 728844 | LOC728844 | -0.6083 | 0.4731 |
| 221938_x_at | 10025 | MED16 | 0.5343 | -0.4156 |
| 222088_s_at | 144195 | SLC2A14 | -0.4524 | 0.3519 |
| 222150_s_at | 54103 | PION | -0.586 | 0.4558 |
| 222160_at | 26993 | AKAP8L | 0.6 | -0.4667 |
| 222201_s_at | 9994 | CASP8AP2 | -0.6124 | 0.4763 |
| 222217_s_at | 11000 | SLC27A3 | 0.6501 | -0.5056 |
| 222326_at | NA | NA | -0.7341 | 0.571 |
| 31874_at | 10634 | GAS2L1 | 0.4683 | -0.3642 |
| 32837_at | 10555 | AGPAT2 | 0.6364 | -0.495 |
| 34689_at | 11277 | TREX1 | 0.6743 | -0.5244 |
| 37462_i_at | 8175 | SF3A2 | 0.4766 | -0.3707 |
| 39729_at | 7001 | PRDX2 | 0.5937 | -0.4618 |
| 41047_at | 79095 | C9orf16 | 0.6252 | -0.4863 |
| 43544_at | 10025 | MED16 | 0.6216 | -0.4835 |
| 50221_at | 7942 | TFEB | 0.6964 | -0.5416 |
| 57715_at | 51063 | CALHM2 | 0.5176 | -0.4026 |
